# Are three weeks hypofractionated radiation therapy (HFRT) comparable to six weeks for newly diagnosed glioblastoma patients? Results of a phase II study

**DOI:** 10.18632/oncotarget.18809

**Published:** 2017-06-28

**Authors:** Pierina Navarria, Federico Pessina, Stefano Tomatis, Riccardo Soffietti, Marco Grimaldi, Egesta Lopci, Arturo Chiti, Antonella Leonetti, Alessandra Casarotti, Marco Rossi, Luca Cozzi, Anna Maria Ascolese, Matteo Simonelli, Simona Marcheselli, Armando Santoro, Elena Clerici, Lorenzo Bello, Marta Scorsetti

**Affiliations:** ^1^ Radiotherapy and Radiosurgery Department, Humanitas Cancer Center and Research Hospital, Rozzano, Italy; ^2^ Neurosurgical Oncology Department, Humanitas Cancer Center and Research Hospital, Rozzano, Italy; ^3^ Consultant of Neurosurgical Oncology Department, Humanitas Cancer Center and Research Hospital, Rozzano, Italy; ^4^ Neuroradiology Unit, Radiology Department, Humanitas Cancer Center and Research Hospital, Rozzano, Italy; ^5^ Nuclear Medicine Department, Humanitas Cancer Center and Research Hospital, Rozzano, Italy; ^6^ Hematology and Oncology Department, Humanitas Cancer Center and Research Hospital, Rozzano, Italy; ^7^ Department of Biomedical Sciences, Humanitas University, Rozzano, Italy; ^8^ Department of Neurology, Humanitas Cancer Center and Research Hospital, Rozzano, Italy; ^9^ Laboratory of Motor Control, Department of Medical Biotechnology and Translational Medicine, Milan University, Milan, Italy

**Keywords:** glioblastoma, hypofractionated radiation therapy, temozolomide, phase II, surgery

## Abstract

**Background:**

The current standard of care for newly diagnosed glioblastoma (GBM) is surgical resection, followed by radiation therapy (RT) with concurrent and adjuvant temozolomide chemotherapy (TMZ-CHT). The patients outcome is still poor. In this study we evaluated hypofractionated radiation therapy (HFRT), instead of standard fractionated radiation therapy, with concomitant and adjuvant TMZ chemotherapy, in terms of safety and effectiveness.

**Methods:**

Patients with newly diagnosed GBM, Karnofsky performance scale (KPS) ≥70, and tumor up to 10 cm underwent maximal feasible surgical resection were treated. HFRT consisted of 60 Gy, in daily fractions of 4 Gy given 5 days per week for 3 weeks. The primary endpoints were overall survival (OS), progression free survival (PFS), and incidence of radiation induced brain toxicity. Secondary endpoint was the evaluation of neurocognitive function.

**Results:**

A total of 97 patients were included in this phase II study. The median age was 60.5 years (range 23-77 years). Debulking surgery was performed in 83.5% of patients, HFRT was completed in all 97 patients, concurrent and adjuvant TMZ in 93 (95.9%). The median number of TMZ cycles was six (range 1-12 cycles). No severe toxicity occurred and the neuropsychological evaluation remained stable. At a median follow up time of 15.2 months the median OS time, 1,2-year OS rate were 15.9 months (95% CI 14-18), 72.2% (95% CI 62.1-80) and 30.4% (95% CI 20.8-40.6). Age, KPS, MGMT methylation status, and extent of surgical resection were significant factors influencing the outcome.

**Conclusion:**

HFRT with concomitant and adjuvant TMZ chemotherapy is an effective and safe treatment.

## INTRODUCTION

The standard of care for newly diagnosed glioblastoma multiforme (GBM) consists of surgical resection, followed by radiation therapy (RT) with concurrent and adjuvant temozolomide chemotherapy (TMZ-CHT). This approach affords a median overall survival (OS) time and a two years OS rate of 14.6 months and 26.5%, respectively [1, 2]. Although, the addition of CHT to RT has led to a survival advantage of two months on the average, the results are still unsatisfactory, and any improvement in this field is mandatory. Increasing evidence indicates that more extensive surgical resection, at least ≥80%, is associated with a longer life expectancy, which become more prominent when the extent of resection (EOR) reaches 95%–100% of the tumor contrast enhancement area [3, 4]. To date, all the attempts to enhance the efficacy of RT were unsuccessful. Dose escalation up to 90 Gy using conventional fractionation or stereotactic radiosurgery boost did not lead to any improvement in outcome, and in most series local recurrence occurred within the high-dose regions [5–10]. The impact of hypofractionated radiation therapy (HFRT) has been investigated as well. The delivery of a higher dose per fraction over a shorter time frame has the advantages to achieve an increase in cells killing and a reduction in accelerated tumor cell repopulation. The initial experiences were carried out in elderly and frail patients with the aim to reduce the overall treatment time in this poor-prognosis subgroup [11–13]. The patients outcome were equivalent to conventional fractionation, although a lower total doses were used. More recently, HFRT has been employed in newly diagnosed GBM patients with a curative aim [14–18]. Retrospective and prospective studies showed that this approach shares similar feasibility and safety results as standard RT schemes, without a growing incidence of neurological toxicity. Nowadays, the impact of HFRT should be investigated in the setting of a multimodality approach which combines concurrent CHT and RT. Consequently, we designed a prospective phase II trial consisting of postoperative HFRT with concurrent and adjuvant TMZ-CHT, following surgical resection, to explore the impact of HFRT on GBM outcome in the modern era. Primary endpoints of the study were overall survival (OS), progression free survival (PFS), and incidence of radiation induced brain toxicity. Secondary endpoint was the evaluation of neurocognitive function.

## RESULTS

### Patients and treatments

From August 2013 to December 2015, out of 125 HGG patients enrolled into the trial, 97 were newly diagnosed GBM. Patients and tumor characteristics are shown in Table 1. Debulking surgery was performed in 80 (82.5%) patients and biopsy in 17 (17.5%). HFRT was carried out in all 97 patients. Concurrent and adjuvant TMZ was performed in 93 (95.9%) patients and omitted in 4 (4.1%) for liver disorders, pulmonary distress, or hematologic toxicity. Characteristics and intensity of treatments are detailed in Table 2. The median follow up time for the whole cohort was 15.2 months (range 3.2-36.8) and 20.2 months (13.1-36.8) for the alive patients.

**Table 1:** Patients and tumor characteristics

	n	%
Patients	97	100
Gender		
Female	36	37
Male	61	63
Age (years)		
Median (range years)	61 (23-74)	
≤60	49	51
61-70	34	35
>70	14	14
KPS		
70	6	6
80	25	26
90	35	36
100	31	32
RPA		
III	8	8
IV	13	14
V	76	78
Tumor molecular profile		
IDH wild type	97	100
MGMT methylated	61	63
MGMT unmethylated	36	37
Number of cerebral lobes involved		
1 lobe	56	58
2 lobes	30	31
3 lobes	5	5
Multifocal tumor	6	6
Median Volumes treated cm^3^ (range cm^3^)		
CTV1	79 (11-197)	
PTV1	166 (34-401)	
CTV2	127 (11-321)	
PTV2	241 (34-510)	
Median max SUV [11C]METPET before HFRT	3·6 (0-8·1)	

KPS=Karnofsky performance scale; RPA=recursive partitioning analysis; IDH1=isocitrate dehydrogenase; MGMT=O-6-methylguanine-DNA methyltransferase; CTV=clinical target volume; PTV=planning target volume; HFRT= hypofractionated radiation therapy; SUV [11C]METPET= 11 carbonione Methionine-Positron Emission Tomography

**Table 2:** Characteristics and intensity of treatments

Variables	n.	%
**Surgery**:	97	100
GTR	53	55
STR	15	15
PR	12	12
Biopsy	17	17
**HFRT:**	97	100
Total doses/dose per fraction Gy		
PTV1	60/4	100
PTV2	42/2·8	100
Number of fractions	15	100
Interruption	0	0
Median duration weeks (range weeks)		3 (2.6-4.1)
**Chemotheraphy:**		
*Concomitant temozolomide*	93	96
Never started concomitant temozolomide	4	4
*Reasons*		
Liver disfunction	2	2
Pulmonary distress	1	1
Hematologic disorder	1	1
*Adjuvant temozolomide*	93	96
Never started adjuvant temozolomide	4	4
*Reasons*		
Hematologic toxicity	2	2
Liver disfunction	2	2
Median number of cycles (range)		6 (0-12)

GTR=gross total resection:<1 cm3 of residual tumor volume; STR=subtotal resection:1-10cm3 of residual tumor volume; PR=partial resection:>10 cm3 of residual tumor volume; HFRT= hypofractionated radiation therapy; PTV=planning target volume

### Progression free survival (PFS) and overall survival analyses

**Figure 1:**
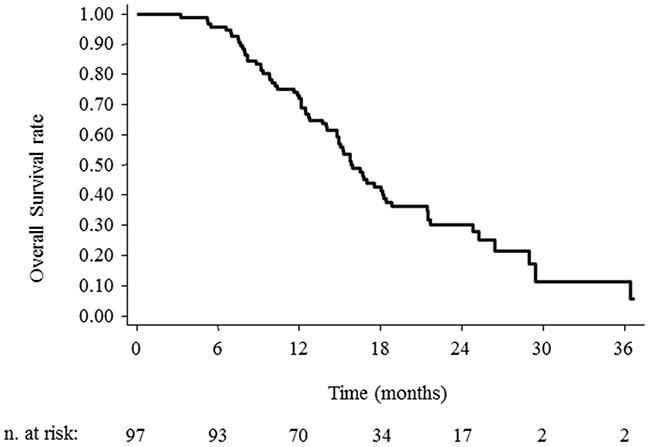
Overall survival for GBM cases treated with hypofractionated radiotherapy with concurrent and adjuvant temozolomide chemotherapy following any entity of surgical resection

**Figure 2:**
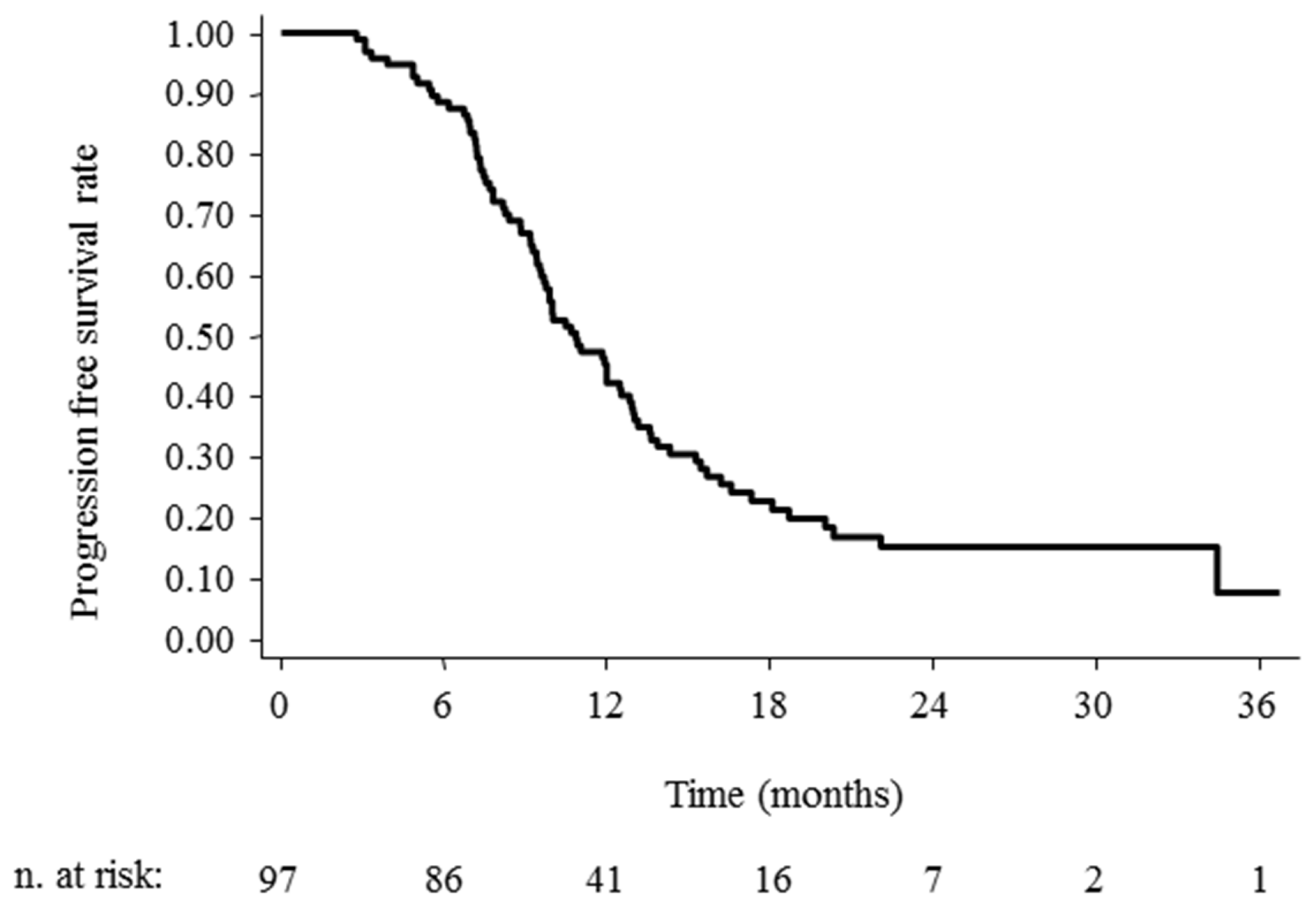
Progression Free Survival (PFS) for GBM cases treated with hypofractionated radiotherapy followed any entity of surgical resection

The median OS time, and the 1, 2-year OS rate were 15.9 months (95% CI 14.8-18.2), 72.2% (95%CI 62.1-80) and 30.4% (95%CI 20.8-40.6) as shown in Figure 1. At the last observation time, 29 (29.9%) patients are alive and 68 (70.1%) dead. The median PFS time, and the 1, 2-year PFS rate were 10.9 months (95% CI 9.6-12.5), 42.3% (95%CI 32.4-51.8), and 15.2% (95% CI 8.2-24.0), respectively as shown in Figure 2.

### Prognostic factors analyses

Survival according to prognostic factors, including age, gender, KPS, MGMT status, EOR, target volume, and number of adjuvant TMZ cycles, was analyzed as well. The highest benefit was observed in treated patients with age ≤60 years, KPS 100, and RPA class 3. Details are shown in Table 3.

**Table 3:** Kaplan-Meyer overall survival according to subgroup analyses

	Patients	MedianOS months(months 95%CI)	1-year OS(95%CI)	2-year OS(95%CI)	p valueunivariate	HRmultivariate(95%CI)	p valuemultivariate
**Overall survival**	97	15.9 (14.8-18.2)	72.2 (62.1-80)	30.4 (20.8-40.6)			
**Gender**							
Female	36	15.2 (10-18.2)	58.3 (40.7-72.4)	23.2 (10.5-38.8)	0.24	0.78	0.36
Male	61	16.7 (14.8-21.7)	80.3 (68-88.3)	34.5 (21.6-47.7)			
**Age**							
≤60	49	18.4 (14.9-24.8)	79.6 (65.4-88.5)	37.3 (23-51.7)	**0.03**	1.39	0.28
>60	48	14.9 (12-16.7)	64.6 (49.4-76.3)	23.2 (11.5-37.2)			
**KPS**							
70	6	9.1 (7.9-ne)	33.3 (4.6-67.6)	0	**<<0**.**01**	0.95	**<0.01**
80	25	12.7 (10-15.8)	68 (46.1-82.5)	13 (2.7-31.8)			
90	35	14.9 (12-16.5)	65.7 (47.6-78.9)	24 (11.1-39.7)			
100	31	25.2 (18.4-ne)	90.3 (72.9-96.8)	56.9 (35.2-73.7)			
**RPA**							
III	8	nr	100	68.6 (21.3-91.2)	**0**.**02**	1.35	0.30
IV	13	15.2 (9.1-ne)	76.9 (44.2-91.9)	26 (6.3-51.7)			
V	76	15.7 (12.7-18)	69.3 (57.6-78.4)	27.5 (17-38.9)			
**EOR**							
GTR	53	17 (15.2-21.4)	81.1 (67.8-89.4)	31.4 (18.1-45.6)	0.13	1.27	**0**.**04**
STR	15	15.9 (9.8-26.4)	73.3 (43.6-89.1)	35.6 (12.1-60.3)			
PR	12	14.8 (8.8-21.5)	75 (40.9-91.2)	13.9 (0.9-44.1)			
Biopsy	17	9.3 (7-ne)	41.2 (18.6-62.6)	29.4 (10.7-51.2)			
**MGMT**							
Methylated	61	18 (14.8-21.7)	75.4 (62.6-84.4)	35.4 (22.7-48.4)	0.07	0.58	**0**.**04**
Unmethylated	36	14.9 (11.8-16.7)	66.7 (48.8-79.5)	21.8 (9.1-38.1)			

KPS= Karnofsky performance scale; RPA=recursive partitioning analysis; EOR=extent of resection;

GTR=gross total resection:<1 cm3 of residual tumor volume; STR=subtotal resection:1-10cm3 of residual tumor volume;

PR=partial resection:>10 cm3 of residual tumor volume; MGMT=O-6-methylguanine-DNA methyltransferase; nr=not reached; ne=not evaluable.

### Postoperative assessment

No mortality or major peri-operative morbidity occurred. Postoperative new neurological deficits were observed in eight (8.2%) patients, in two cases recovered during RT treatment; they consisted in motor deficit in three, hemianopsia in two, aphasia in two, and motor deficit plus hemianopsia in one patient.

### CHT-HFRT side effects

All patients completed the scheduled HFRT plan; a transient neurological deterioration was recorded in two patients, consisting of partial seizure and aphasia. Grade I-II radionecrosis (RN) occurred in 22 (22.7%) patients. No Grade III- IV radionecrosis were observed. Fatigue was observed in 55 (56.7%) patients during concurrent CHT-HFRT. Grade 2-4 hematologic toxicity was recorded in 12 (12.9%) patients, in three during concomitant treatment and in nine patients during adjuvant TMZ. No thromboembolic events or cerebral hemorrhage occurred.

### Neuropsychological evaluation

lNeuropsychological scores before and after HFRT remained unchanged. The analysis showed no detrimental effect of HFRT on cognitive functions (language, short and long term verbal and visuo-spatial memory, working memory, attentive and executive functions). Particularly, a significant performance improvement was detected: between T0 and T1 in the copy of the figure test (p value<<0.01), in the recall of the complex figure tests (p value <<0.01), in the ideomotor apraxia test (p=0.03), and in the trail making test part A (p value=0.04); between T1 and T2 in the ideomotor apraxia test (p value=0.02) and in the trail making test part B-A (p value=0.01) (Table 4).

**Table 4:** Neuropsychological outcome

Cognitive domains	Test	T0		T1			T2			T3		
		Before HFRT		1 month after HFRT			6 months after HFRT			12 months after HFRT		
		n.	mean	n.	mean	p value(T0-T1)	n.	mean	p value(T0-T2)	n.	mean	p value(T0-T3)
**Language**	Token	85	29.1	80	29.7	0.1	33	28.5	0.2	12	28.5	0.50
	Picture Naming	85	42.2	85	43.7	0.07	33	43.5	0.43	12	42.9	0.71
	Fonological fluency	75	20.5	73	22.6	0.12	31	20.3	1.0	11	20.2	0.95
	Semantic Fluency	74	29.7	73	32.6	0.05*	31	32.1	0.13	11	32.7	0.21
**Memory**	Digit span forward	85	5.0	79	5.1	0.31	32	4.9	0.68	11	4.8	1.00
	Corsi span forward	85	4.0	80	4.1	0.22	34	4.3	0.18	11	4.0	0.75
	Digit span backward	85	3.2	79	3.4	0.14	33	3.5	0.93	11	3.4	0.63
	Corsi span backward	84	3.5	80	3.7	0.11	32	3.7	0.80	12	3.4	0.45
	Rey's 15 words test - immediate	72	29.7	67	30.4	0.42	29	29.8	0.32	11	31.8	0.29
	Rey's 15 words test - delayed	72	5.0	66	5.1	0.96	28	5.1	0.53	11	5.5	0.55
	Recall complex figure	78	9.8	78	12.0	<<0.01*	32	13.4	0.01	12	12.9	0.20
**Visuo-constuctional abilities**	Copy of the figure	78	25.7	78	28.1	<<0.01*	31	27.7	0.49	12	29.3	0.24
**Apraxia**	Ideomotor apraxia	85	66.0	79	66.2	0.03*	31	67.4	0.02*	12	56.2	0.33
	Orofacial apraxia	84	19.2	78	19.4	0.16	31	19.3	0.30	12	19.2	0.32
**Attention and executive functions**	Trail Making test - a	34	54.9	38	50.1	0.04*	23	52.7	0.78	8	44.6	0.32
	Trail Making test - b	31	169.8	37	168.8	0.08	21	157.8	0.06	6	150.2	0.18
	Trail Making test - b-a	31	114.8	37	123.5	0.26	21	105.9	0.01*	6	108.1	0.18
	Attentive matrices	84	41.3	80	41.6	0.37	33	40.7	0.70	12	37.4	0.08
	Stroop Test - error	70	2.0	72	1.2	0.30	28	1.8	0.62	10	2.9	0.95
	Stroop Test - time	70	28.7	72	28.9	0.56	28	35.1	0.71	10	52.3	0.41
	Raven coulored progressive matrices	80	26.3	74	27.2	0.24	31	27.6	0.22	10	29.1	0.47

HFRT=hypofractionated radiation therapy; N=number of patients; mean= age-education-adjusted score for each test and for each time point;

T0: before HFRT; T1:1 month after HFRT; T2:6 months after HFRT; T3:12 months after HFRT; * significant p values (p ≤ 0·05)

### Treatment at progression

Brain progression occurred in 77 (79.4%) patients and a salvage treatment was performed in 34 (44.2%). It consisted in surgery alone in one, radiation therapy alone in one, surgery with sequential chemo-radiotherapy in five and second line chemotherapy alone in 26. The chemotherapeutic agents more frequently used were fotemustine and temozolomide. The median survival time from progression was 8.5 months (95% CI 5.4-11.4).

## DISCUSSION

Based on the results of the phase III EORTC-NCIC trial, the current standard treatment for newly diagnosed GBM patients consists of radiotherapy with concomitant and adjuvant TMZ-CHT [1]. Fractionated conformal three-dimensional radiotherapy (3DCRT) to a total dose of 60 Gy in 30 daily fractions of 2 Gy each is employed based on the results from previous dose exploratory studies [19]. The use of protracted RT schedules harbors the theoretical drawback of allowing a cell repopulation, which could be of relevance in tumors with a rapid doubling time such as GBM [20]. This effect may be seen in routine clinical practice as well, where a widely rate of patients, up to 10%, discontinues RT for disease progression [1]. Hypofractionated radiation therapy (HFRT) offers the advantages of achieving an increased cells killing action, by the delivery of a higher dose per fraction over a shorter time frame, and of reducing the effect of accelerated tumor cell repopulation by shrinking the RT treatment time. Based on these observations, we designed this phase II study to assess the feasibility and the effectiveness of HFRT schedule within a multimodal therapeutic approach, including concurrent and adjuvant TMZ-CHT, following surgical resection. Although the initial study design included all grade of HGG, we report here the analysis of the newly diagnosed IDH wild type GBM patients only, to deal with a highly homogeneous population. To now, few data are available in GBM patients on the impact of HFRT used to radical doses into a multimodal strategy including surgery, concomitant and adjuvant chemotherapy [14–18]. The published studies are often phase I investigation, with a limited number of cases treated, quite heterogeneous for patients and tumors characteristics. However, the preliminary results were promising, with a median OS up to 20 months in some series, and with a low incidence of symptomatic radionecrosis or severe neurologic side effects. Table 5 showed some of the most relevant phase I-II studies about this issue. There is no agreement about the optimal HFRT doses to deliver and the schedule to employ. With the aim to improve the impact on outcome, a therapeutic effective dose greater than that of conventional radiotherapy (BED_10_ 84Gy vs 72Gy) was applied. Our results compare favorably with previous reports regarding standard RT treatment [1], with a median OS time, 1, 2-year OS rate of 15.8 months, 72.2% and 28.5%, and a median PFS time, 1, 2-year PFS rate of 10.8 months, 42.3% and 16%, respectively, as shown in Table 6. Employing this approach, notwithstanding a higher dose on large tumor volume was delivered, all patients completed HFRT treatment, no neurological deterioration was observed and neurocognitive functions remained stable or in some cases improved, as shown by neuropsychological evaluation. Published data suggest an increased incidence of grade III-IV radionecrosis when a high dose per fraction and therapeutic effective doses are used, ranging from 3% to 20% according to the various scheme utilized [21, 22]. In our analysis only grade I-II toxicity was recorded and in no patients grade III-IV was detected. Several prognostic factors were investigated. As in many other published studies, in our series age was a factor strongly influencing survival (p=0.01). The greater benefit of treatment was observed in patients younger than 60 years, with a percentage of 40% alive at two years. Along with age, KPS was found to significantly affect outcome as well, with more than 50% of patients with KPS 100 surviving beyond two years (p=<<0.01). The extent of surgical resection has been commonly considered as a factor affecting outcome in GBM patients [3, 4, 23]. Different methodologies to define the amount of tumor removal have been used. In our study, we defined three groups of patients in relation to the RTV. A favorable trend was observed in case of RTV <10 cm^3^ that was obtained in about 70% of patients (p=0.03). Although an extensive surgical resection was performed in the largest number of patients treated, no major perioperative morbidity occurred and adjuvant treatments was started without delays. Literature data proved that postoperative neurological functions are crucial elements for patient quality of life (QoL) and OS. As reported by McGirt et al, new motor or language deficits are associated with significant decreases in the median survival of patients with GBM supporting the need of coupling maximal tumor resection with preservation of neurological integrity [24]. Considering the small size of RTV and the absence of major neurological deficit, no increase in corticosteroid drugs or AED dosage was needed during concomitant chemo-radiotherapy, and the HFRT was well tolerated along all treatment period. One might speculate that a radiotherapy intensification jeopardizes the patient ability of completing an adequate adjuvant chemotherapy treatment. In our cohort, TMZ was successfully administered to 96% of patients, and more than 50% of them received more than 6 cycles. In addition, salvage treatment at progression was done in about 45% of patients and the median survival of patients was 8.5 months, clearly higher respect to other series [1, 2]. We are aware that our study does not reach the power of a randomized trial in terms of sample size and longer follow up time. Nevertheless, the results shown indicates that HFRT is a feasible and safe approach, affording good survival rates, and considerable decrease in the treatment time (from 6 to 3 weeks), which could be relevant in a group with short life expectancy, such as GBM patients. Subjects who had the greatest benefit are those with young age, good performance status, maximal safe resection, and a MGMT methylated tumor. Open questions remain the optimal radiation total doses to deliver and the schedule to utilize, but HFRT within a multimodal therapeutic approach, seems a way forward to improve the outcome of patients with glioblastoma.

**Table 5:** Published studies regarding hypofractionated radiation therapy

Authors	N pts	Study design	Concurrent and adjuvant TMZ	Total dose	N frs	RN	Median OS	1 yearOS %	2 yearsOS %
Chen (14)	16	Phase I	yes	60 Gy	20151210	1 G43 G3	16.3 months	nr	nr
Terasaky (16)	26	Pilot	yes	45 Gy	15	no	15.6 months	nr	nr
Reddy (15)	24	Phase II	yes	60 Gy30 Gy	1010	no	16.6 months	nr	nr
Jastaniyah (17)	25	Phase I	yes	60 Gy	22	no	15.7 months	62	9
Iuchi (18)	46	Phase II	yes	68 Gy40 Gy32 Gy	888	20 G3	20 months	50	41

Pts=patients; TMZ=temozolomide; frs=fractions; RN=radionecrosis; OS=overall survival; no=not observed; nr=not reported

**Table 6:** comparison between standard (6 weeks) radiation therapy (Stupp et al. [1]) and hypofractionated (3 weeks) radiation therapy within multimodal approach

Variables	Concurrent and adjuvant TMZ CHTwith standard RT(60 Gy in 30 fractions)		Concurrent and adjuvant TMZ CHTwith hypofractionated RT(60 Gy in 15 fractions)	
***Extent of surgery***				
Biopsy	17%		17.5%	
Debulking	83%		82.5%	
*Radiotherapy*				
Never started	1%		0%	
Early discontinuation*	10%		0%	
*Concurrent TMZ CHT*				
Never started	2%		4.1%	
*Adjuvant TMZ CHT*				
Never started	22%		4%	
Number of median cycles (range)	3 (0-7)		6 (0-12)	
*PFS*	(95%CI)		(95%CI)	
Median	6.9 months	(5.8-8.2)	10.9 months	(9.6-12.5)
1year	26.9%	(21.8-32.1)	42.3%	(32.4-51.8)
2 year	10.7%	(7.0-14.3)	15.2%	(8.2-24.0)
*OS*				
Median	14.6 months	(13.2-16-8)	15.9 months	(14.8-18.2)
1 year	61.1%	(55.4-66.7)	72.2%	(62.1-80)
2 year	26.5%	(21.2-31.7)	30.4%	(20.8-40.6)

* for disease progression

TMZ=temozolomide; CHT=chemotherapy; PFS=progression free survival; OS= overall survival

## MATERIALS AND METHODS

### Study design and patients

The present trial was a prospective single arm phase II study approved by our institutional review board. The trial was registered at ClinicalTrials.gov site with number NCT00006353. All patients provided a written informed consent to the treatment and the use of their data for scientific purposes. Eligible patients had: 1) an age of 18-70 years and a Karnofsky performance scale (KPS) ≥60; 2) a newly diagnosed high grade gliomas (HGG); 3) a residual tumor or surgical cavity with a maximum diameter of 10 cm; 4) a normal liver, kidney and bone marrow functions. A limited number of patients older than 70 years with KPS ≥80 were included as well. We report here the analysis of GBM patient population group only.

### Procedures

#### Surgery

Surgery was performed in all patients with the aim to maximally remove the tumor according to functional boundaries. Tumor removal was achieved with the aid of brain mapping techniques and imaging neuro-navigation (post contrast T1 weighted images, MET-PET, FLAIR, functional MRI, DTI) coupled with intraoperative ultrasounds, to afford maximal resection and maintenance of full patient functional integrity. Extent of resection (EOR) was determined by comparing preoperative post-contrast T1 weighted MRI with postoperative MRI study, acquired within 48 hours after surgery, and calculated as follows: preoperative tumor volume – postoperative tumour volume/preoperative tumor volume. Gross total resection (GTR) was defined as residual tumor volume (RTV) lower than 1 cm^3^, subtotal resection (STR) when RTV was among 1 and 10 cm^3^, and partial resection (PR) when it was greater than 10 cm^3^ [25]. Patients who received biopsy only were also enrolled. Tumor molecular profile was available in all cases. Immunohistochemical staining for Isocitrate dehydrogenase (IDH1/2) was performed on BenchMark XT automated tissue staining systems (Ventana Medical Systems, Inc., Tucson, AZ) using validated protocols. O-6-methylguanine-DNA methyltransferase (MGMT) promoter methylation status was determined by pyrosequencing (Diatech Pharmacogenetics, MGMT plus, valid CE/IVD) [26].

#### Hypofractionated radiation therapy (HFRT)

CT scan, T1-weighted FLAIR (fluid-attenuated inversion recovery images) and T2-weighted 3D-FLAIR followed by T1-weighted MPRAGE MRI and [11C]-Methionine-PET (11CMETPET) were acquired for radiation therapy planning and images were co-registered each other. Pre and post-operative MRI acquired within 48 hours from surgery were used too to better define the target RT volume. Two different clinical target volume (CTV) were outlined: CTV1 corresponded to the entire surgical cavity plus eventual residual tumor after surgery or, to the abnormality on the T1-weighted post-contrast MPRAGE and 11CMETPET in case of biopsy; CTV2 corresponded to the abnormality on FLAIR MRI images before surgery. Planning target volume 1-2 (PTV1/PTV2) was generated adding an isotropic margin of 5 mm from CTV1 and CTV2 respectively. Intensity modulated radiation therapy was performed within 4-6 weeks after surgery using volumetric modulated arc therapy (VMAT). The dose prescribed was 60 Gy with daily fraction of 4 Gy on PTV1, and 42 Gy with daily fraction of 2.8 Gy on PTV2 for 15 consecutive days, using a simultaneous integrated boost (SIB). Organs at risk (OARs) outlined were optic nerves and chiasm, lens, brainstem and cochlea without additional margins, and the recommended maximal doses were ≤40 Gy, ≤10 Gy, ≤30 Gy, and ≤30 Gy, respectively. Dose was prescribed to an isodose line that ensured that more than 98% of PTV1-2 receives 95% of prescribed dose. In each session, patients position check was performed using ExacTrac (Brain Lab) system and cone beam computer tomography (CBCT).

#### Chemotherapy

All patients received TMZ concurrently with HFRT. TMZ was administered orally, once daily, at 75 mg/m2, starting on the first day of HFRT and continuing for the whole treatment. After a 4-week break adjuvant TMZ was administered at 150 to 200 mg/m^2^ orally, once daily, for 5 consecutive days every 28 days up to 12 cycles, or until disease progression occurred.

#### Supportive care

Corticosteroids were administered during the whole HFRT treatment and progressively reduced at the end of RT. Antiepileptic drugs (AEDs) were prescribed only in patients with a history of at least one seizure. The most frequently used AEDs were levetiracetam as first line instance followed by topiramate, lamotrigine or lacosamide.

### Evaluation of clinical outcome

Clinical outcome was evaluated by neurological examination and MRI imaging 1 months after concurrent CHT-HFRT and every four months thereafter. [11C]MET-PET was performed at four and 12 months during maintenance CHT or to rule out pseudo-progression. When needed tumor progression was defined according to Response Assessment in Neuro-Oncology (RANO) working group [27]. Thirty-days postoperative morbidity and mortality were assessed. Major complications were defined as the appearance of new neurological deficits persisting for more than 30 days after surgery and requiring a prolonged hospitalization or rehabilitation. All other complications were defined as minor. The appearance of new deficits or the worsening of preoperative deficits were considered as complications. Hematologic and non-hematologic toxicities, including radionecrosis, were graded according to Common Terminology Criteria for Adverse Events version 3.0. Neuropsychological assessment was performed through a shortened version of the “Milano Bicocca Battery”, evaluating language, memory, apraxia, visuo-constructional abilities, attentive and executive functions [28]. The total time of administration was one hour on average. Patients were evaluated one week before the start of HFRT (T0), 1 (T1), 6 (T2) and 12 months (T3) thereafter or until disease progression.

### Statistical analysis

The study was designed to demonstrate, with 90% power and 1 sided α error of 5%, a significant difference between a reference value of 1 year survival of 55.2% in standard treatments compared to a figure of 71.4% for the new one. Variables considered were: gender, age, KPS status, RPA class, EOR and MGMT methylated status. Age of patients was divided into two groups, respectively, ≤ 60 and >60 years. Survival and recurrence time observations were evaluated according to the method of Kaplan and Meier, starting from the date of diagnosis. The median survival time is evaluated obtained from Ŝ(t), the Kaplan –Meier product-limit estimate of the survivor function. Confidence bounds of the survivor function are calculated based on the asymptotic variance of ln[−ln Ŝ(t), as described in Kalbfleisch and Prentice. The upper (lower) confidence limits for the median survival times are defined as the first time at which the upper (lower) confidence limit for Ŝ(t) is less than or equal to 0.5. A not reached indicator (nr) was specified if the survival estimate resulted above the 50% level in the considered observation time. Upper confidence bound of median survival time was labeled as ne if not evaluable with the above method for a specific group of patients in the considered time of observation. In order to assess the prognostic role of the different individual variables, the log-rank test was used for dichotomous variables gender, MGMT and age (grouped). Univariate cox model was applied for the remaining variables. Multivariate Cox regression model was used as a method to estimate the independent association of our variable set with overall survival. Neurophysiological assessment parameters were preliminarily evaluated for a difference before and after RT. In order to assess the effect of the HFRT on neurocognitive functions a series of nonparametric analyses (Wilcoxon signed-rank test) was performed. P value and the mean of the age- and education-adjusted score for each test and for each time point (T0;T1;T2;T3) was reported. Statistical analysis was performed by the use of the Stata software, version 13.1 (Stata Corp LP, College Station TX USA). Methods and calculation details can also be found in the software refence manual in the stata website (http://www.stata.com/).

## Abbreviations

GBM: glioblastoma multiforme
RT: radiotherapy
CHT: chemotherapy
EOR: estent of resection
TMS: temozolomide
HFRT: hypofractionated radiaton therapy
KPS: Karnofsky performance scale
OS: overall survival
PFS: progression free survival
GTR: gross total resection
RTV: residual tumor volume
STR: subtotal resection
PR: partial resection
MET-PET: methionine PET
FLAIR: Fluid-attenuated inversion recovery
MRI: magnetic resonance imaging
DTI: diffusion tensor imaging
IDH: isocitrate dehydrogenase
MPRAGE: magnetization-prepared rapid gradient-echo
11CMETPET: [11C]-Methionine-PET
MGMT: O-6-methylguanine-DNA methyltransferase
CTV: clinical target volume
PTV: planning target volume
VMAT: volumetric modulated arc therapy
SIB: simultaneous integrated boost
OAR: organ at risk
CBCT: cone beam computed tomography
AED: antiepileptic drugs
RANO: Response Assessment in Neuro-Oncology
RPA: recursive partiotioning analysis
